# *In Vitro* Antimicrobial Activity Screening of Ethanol Extract of *Lavandula stoechas* and Investigation of Its Biochemical Composition

**DOI:** 10.1155/2019/3201458

**Published:** 2019-01-10

**Authors:** Kerem Canlı, Ali Yetgin, Atakan Benek, Mustafa Eray Bozyel, Ergin Murat Altuner

**Affiliations:** ^1^Department of Biology, Faculty of Science, Dokuz Eylül University, Izmir, Turkey; ^2^Department of Biotechnology, Institute of Engineering and Science, Izmir Institute of Technology, Izmir, Turkey; ^3^Department of Biology Education, Buca Faculty of Education, Dokuz Eylül University, Izmir, Turkey; ^4^Department of Biology, Faculty of Arts and Science, Canakkale Onsekiz Mart University, Canakkale, Turkey; ^5^Department of Biology, Faculty of Science and Arts, Kastamonu University, Kastamonu, Turkey

## Abstract

The aim of this study was to test antimicrobial activity of ethanol extract of *Lavandula stoechas* against 22 bacteria and 1 yeast. Also, biochemical composition of the extract was investigated. A wide range of Gram-positive, Gram-negative microorganisms, and multidrug resistant bacteria were selected to test the antimicrobial activity. As a result, the extract is observed to contain fenchone *(bicyclo[2.2.1]heptan-2-one, 1,3,3-trimethyl-, (1R*)-) and camphor *(+)-2-bornanone)* as major components and showed antimicrobial activity against all studied microorganisms except *Escherichia coli* ATCC 25922 and *Klebsiella pneumoniae*. The results of the study present that *L. stoechas* is active against MDR strains too.

## 1. Introduction

The World Health Organization (WHO) has predicted increasing antimicrobial resistance as a major threat for the public health for the twenty-first century. In order to prevent spreading of antibiotic resistance infections, scientists have been conducting intensive researches to determine new antimicrobial agents. One way to prevent antibiotic resistance of microorganisms is by using new compounds that are not based on existing antimicrobial agents.

The *Lavandula* genus is an important member of family Lamiaceae. It consists of 47 species of small evergreen shrubs having aromatic foliage and flowers [1]. *Lavandula* species are cultivated in France, Spain, and Italy. In Turkey, mainly two species, *Lavandula stoechas* and *Lavandula angustifolia*, and their subspecies and hybrid forms grow wildly or are cultivated [2]. The medicinal importance of the plant is well documented [3, 4], and the drugs prepared from this plant are registered in many Pharmacopeia [5]. *L. stoechas* L. is used in perfumery and cosmetics [6, 7]. Anticonvulsant, sedative, and antispasmodic activities were reported [8]. The essential oil (EO) of *L. stoechas* possesses weak antibacterial activity [9]. It is used in folk medicine as an antispasmodic, a sedative, and a diuretic and for rheumatic diseases [7]. The main purpose of this study was to investigate the antimicrobial activity of *L. stoechas* and reveal the major components of its ethanol extracts.

## 2. Materials and Methods

### 2.1. Endemic Plant Samples

Dried flowers of *L. stoechas* L. were purchased from the local market in Canakkale, Turkey, and identified by Dr. Mustafa Eray Bozyel.

### 2.2. Disk Diffusion Test

Plant samples were dried after collection and ground into small pieces with a grinder. Ground *L. stoechas* samples were shaken in ethanol (Sigma-Aldrich) at 125 rpm for 2 days at room temperature. After that, all the mixture was filtrated through Whatman no. 1 filter paper into evaporation flasks. Filtrates were evaporated by a rotary evaporator (Buchi R3) at 45°C [10, 11]. Finally, the remnants were collected and weighed. 5.83, 23.4, and 35.1 mg samples were prepared. The activity of the extract was tested against 22 bacteria and 1 yeast, where most of the strains were standard; nostandard strains were isolated from food and the MDR strains were clinical isolates. Nonstandard strains were identified in Ankara University, Department of Biology and Duzce University, Department of medical. All bacterial strains were incubated at 37°C for 24 hours; however, *Candida albicans* was incubated at 27°C for 48 hours [11]. Each bacteria and yeast were inoculated into 0.9% sterile saline solution and adjusted to 0.5 McFarland standard, in order to standardize inocula to contain about 10^8^ cfu·mL^−1^ for bacteria and 10^7^ cfu·mL^−1^ for *C. albicans* [11]. The antimicrobial activity of ethanol extract of *L. stoechas* was tested by the disk diffusion test, as mentioned before [12]. Firstly, Mueller–Hinton agar (BD Difco, USA) was poured into 90 mm sterile Petri dish in order to reach a meant depth of 4.0 mm ± 0.5 mm. The extracts were loaded on 6 mm Oxoid Antimicrobial Susceptibility Test Disks. Disks were left to dry overnight at 30°C in sterile conditions in order to prevent any remaining of solvent, which may interfere with the results. After that, prepared microorganisms, which were inoculated into saline solution, were streaked on the surface of Petri dishes. These plates were left to dry for 5 minutes at room temperature in aseptic conditions [12]. Next, disks were tightly applied to the surface of plates. Finally, these plates were incubated, and inhibition zone diameters were recorded [12].

Preculturing conditions for all microorganisms are as mentioned previously[13].

### 2.3. Broth Dilution Test

Broth dilution method for minimum inhibitory concentration (MIC) determination as described previously was employed [14]. Serial 2-fold dilutions were made to obtain a concentration range of 0.07–35.9 *µ*g/mL. The MIC was defined as the lowest concentration of extract inhibiting any visible bacterial growth. All tests were conducted in triplicates.

### 2.4. GC-MS Analysis

For the identification of chemical components, each sample was analyzed by Agilent GC 6890N-Agilent MS 5973 equipped with HP5-MS capillary column (30m ∗ 0.25 mm; coating thickness 0.25 *μ*m). Analytical conditions were an injector temperature of 350°C; carrier gas helium at 1 mL/min; injection mode: split, split ratio 10 : 1; volume injected: 1 *μ*L of sample in ethanol extract; oven temperature programmed from 40°C to 350°C at 4°C/min; pressure: 48.2 kPa; and split flow: 9.9 mL/min. The MS scan conditions were a transfer line temperature of 280°C, an interface temperature of 280°C, and an ion source temperature of 230°C. Identification of the components was conducted by matching the retention times against National Institute of Standards and Technology (NIST Mass Spectrometry DATA CENTER) data library, and crosscheck was applied with previously published data [15, 16] The chemical components found to be higher than 1% were accepted as the major components, and the list of these components and information regarding them are given in Table 1.

### 2.5. Controls

Empty sterile disks and extraction solvent (ethanol) were used as negative controls. Ciprofloxacin and gentamicin used as pozitif controls (Table 2).

### 2.6. Statistics

The statistical analysis was executed using a parametric method, the one-way analysis of variance (ANOVA), with a significance level of 0.05. In order to put forward any correlation between concentration and antimicrobial activity, Pearson correlation coefficient was calculated. All statistical analysis were conducted by using R Studio, version 3.3.2 [17].

## 3. Results and Discussion

The diameters of inhibition zones, which were measured in millimeters, are given in Table 3 as the mean values of three parallels with standard errors. No activities were observed for the negative controls. Furthermore, statistical analysis proved that there are no significant differences between the activities of three parallels of each extract volumes (*p* > 0.05). On the other hand, a weak positive correlation is observed between the activities of extracts and the volumes tested, with a Pearson correlation coefficient of 0.3239.

In addition, the results of broth dilution test (MIC values) are given in Table 4.

According to Table 3, *L. stoechas* has antimicrobial activity against all studied microorganisms except *Escherichia coli* ATCC 25922 and *Klebsiella pneumoniae*. Three of them have high susceptibility (15–25 mm); seven of them have moderate susceptibility (14–10 mm); and eleven of them have low susceptibility (9–7 mm). *L. stoechas* shows antimicrobial activity against all tested MDR bacteria. These results are important since antimicrobial activity of this species were determined against large range of Gram-negative and Gram-positive bacteria.

**Figure 1:**
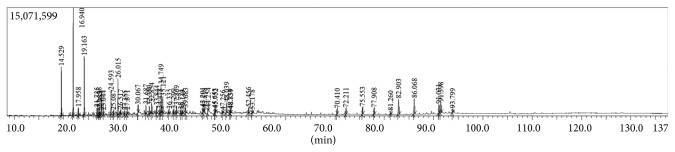
GC-MS chromatography of *L. stoechas*.

According to GC-MS results, fenchone *(bicyclo[2.2.1]heptan-2-one, 1,3,3-trimethyl (1R)-)*, and camphor *(+)-2-bornanone)* are mainly found in the composition of *L. stoechas* ethanol extract. Similar results were obtained when compared with previous researches [18–20] (Figure 1).

The antimicrobial effect of the plant is known from previous investigations, but there is no broad-spectrum study like this [18, 20]. It has also been reported for the first time that the plant's ethanol extract is effective against multidrug-resistant microorganisms, which is one of the most important health hazards in the world [21].

Acinetobacter species, particularly *Acinetobacter baumannii*, have become significant pathogens especially in the nosocomial setting. *A. baumannii* has progressively been implicated in serious nosocomial infections, including bloodstream infection (BSI), nosocomial and ventilator-related pneumonia, and meningitis. These infections are particularly common in critically ill patients, with mortalities as high as 40–64% for pneumonia and 17–46% for BSI [22]. The extensive use of broad-spectrum antibiotic agents within hospitals has led to the rapid emergence of multidrug-resistant (MDR) *A. baumannii* strains. Only a few antimicrobial agents are active against MDR *A. baumannii* infections [22]. In our study, we observed 11 mm of inhibition zone against *A. baumannii* (MDR) strain. Moreover, we have found 14 mm of inhibition zones against *Proteus vulgaris* (MDR) and *Streptococcus pneumoniae* (MDR) strains. Our results present that *L. stoechas* is active against MDR strains too.

Previous studies tested the antimicrobial activity of *L. stoechas* and found MIC values between 0 and 6500 *µ*g/mL [23]. When the result of this study were compared with the results of the previous study, it may be observed that the MIC values obtained in our study were much lower, which is better. The reason of this difference should be related with the strains used in two studies. Although *B. subtilis*, *S. aureus*, *S. typhimurium*, *E. coli*, and *C. albicans* were used in both studies, the strains were different. Only *L. monocytogenes* ATCC 7644 were common in these two studies but the MIC values were different. This difference could be related with the composition of extracts which were directly affected by the environment; those plant samples were collected.

Another previous study was tested the antimicrobial activity of two subspecies of *L. stoechas*. The only common microorganism used in our study and this previous study was *C. albicans*. They observed a MIC value > 100 *µ*g/mL for both subspecies, which are quite higher than our results [24]. But this difference is logical since the plant samples, *C. albicans* strain, and collection area were different.

Ez Zoubi et al. [25] tested the antimicrobial activity of the essential oil of *L. stoechas* against *E. coli*, *K. pneumoniae*, *Proteus mirabilis*, and *P. aeruginosa*. As a result, they observed MIC values between 2.5 and 10 *µ*g/mL. Although the strains of these microorganisms were not defined, the difference in the MIC values between this study and our study was mainly due to using different types of extracts, namely, essential oil and ethanol extracts.

## 4. Conclusion

Our study clearly presents that *L. stoechas* should have a possible medicinal uses, especially against MDR bacteria. However, further researches are needed in order to analyse the active substances and their activity mechanisms in details.

## Figures and Tables

**Table 1:** The major chemical components of *L. stoechas* according to the GC-MS analysis.

No.	Retention time	Compound name	Formula	Molecular weight (g/mol)	Area (%)
1	14.529	Eucalyptol	C_10_H_18_O	154.249	6.22
2	16.940	Bicyclo[2.2.1]heptan-2-one, 1,3,3-trimethyl-, (1R)-	C_10_H_16_O	152.233	18.81
3	19.163	(+)-2-Bornanone	C_10_H_16_O	152.233	8.64
4	22.138	Fenchyl acetate	C_12_H_20_O_2_	196.286	1.10
5	24.593	Bornyl acetate	C_12_H_20_O_2_	196.286	3.40
6	26.015	Myrtenyl acetate	C_12_H_18_O_2_	194.270	5.18
7	30.067	Benzaldehyde, 2-hydroxy-4-methyl-	C_8_H_8_O_2_	136.148	1.64
8	31.687	Cubedol	C_15_H_26_O	222.366	1.10
9	32.350	Cubedol	C_15_H_26_O	222.366	1.08
10	32.854	Bicyclo[3.1.1]hept-3-en-2-one, 4,6,6-trimethyl-	C_10_H_14_O	150.218	1.40
11	33.844	Acetic acid, 4a-methyl-1,2,3,4,4a,5,6,7-octahydronaphthalen-2-yl ester			1.42
12	34.749	Veridiflorol	C_15_H_26_O	222.366	4.50
13	35.121	Viridiflorol	C_15_H_26_O	222.366	2.99
14	37.869	Unknown	—	—	1.02
15	39.683	Andrographolide	C_20_H_30_O_5_	350.449	1.15
16	48.039	Palmitic acid	C_16_H_32_O_2_	256.424	2.48
17	52.456	Behenic alcohol	C_22_H_46_O	326.600	1.06
18	75.553	Tetrapentacontane	C_54_H_110_	759.451	1.48
19	77.908	Tetrapentacontane	C_54_H_110_	759.451	1.37
20	82.903	Tetrapentacontane	C_54_H_110_	759.451	4.84
21	86.068	Tetrapentacontane	C_54_H_110_	759.451	4.83
22	91.031	Tetrapentacontane	C_54_H_110_	759.451	2.53
23	91.398	Stigmast-5-en-3-ol, (3.beta.)-	C_29_H_50_O	414.707	3.25

**Table 2:** Pozitif controls (inhibition zones in mm).

	Ciprofloxacin	Gentamicin
*B. subtilis* DSMZ 1971	36	30
*C. albicans* DSMZ 1386	—	—
*E. aerogenes* ATCC 13048	30	23
*E. durans*	24	14
*E. faecalis* ATCC 29212	19	13
*E. faecium*	28	28
*E. coli* ATCC 25922	—	20
*K. pneumoniae*	30	22
*L. innocula*	18	13
*L. monocytogenes* ATCC 7644	20	28
*P. aeruginosa* DSMZ 50071	28	15
*P. fluorescens* P1	19	12
*S. enteritidis* ATCC 13075	36	24
*S. infantis*	24	24
*S. Kentucky*	34	13
*S. typhimurium* SL 1344	35	23
*S. aureus* ATCC 25923	22	24
*S. epidermidis* DSMZ 20044	34	25

—: no activity observed.

**Table 3:** Disk diffusion test result for *L. stoechas* (inhibition zones in mm).

	5.83 mg loaded disk	23.4 mg loaded disk	35.1 mg loaded disk
*Bacillus subtilis* DSMZ 1971	11.00 ± 0.00	13.00 ± 0.00	14.00 ± 0.58
*Candida albicans* DSMZ 1386	7.00 ± 0.00	9.00 ± 0.00	11.00 ± 0.00
*Enterobacter aerogenes* ATCC 13048	—	—	8.00 ± 0.00
*Escherichia coli*	—	—	8.00 ± 0.00
*Escherichia coli* ATCC 25922	—	—	—
*Enterococcus durans*	—	10.00 ± 0.00	11.00 ± 0.00
*Enterecoccus faecalis* ATCC 29212	7.00 ± 0.00	9.00 ± 0.00	11.00 ± 0.00
*Enterecoccus faecium*	11.00 ± 0.58	16.00 ± 0.58	15.00 ± 1.15
*Klebsiella pneumoniae*	—	—	—
*Listeria innocua*	—	8.00 ± 0.00	10.00 ± 0.00
*Listeria monocytogenes* ATCC 7644	9.00 ± 0.00	14.00 ± 1.15	13.00 ± 0.58
*Pseudomonas aeruginosa* DSMZ 50071	—	8.00 ± 0.00	10.00 ± 0.00
*Pseudomonas fluorescens* P1	7.00 ± 0.00	9.00 ± 0.00	13.00 ± 0.00
*Staphylococcus aureus*	10.00 ± 0.00	10.00 ± 0.00	14.00 ± 0.58
*Staphylococcus aureus* ATCC 25923	12.00 ± 0.58	13.00 ± 0.00	18.00 ± 0.00
*Salmonella enteritidis* ATCC 13076	7.00 ± 0.00	9.00 ± 0.00	10.00 ± 0.00
*Staphylococcus epidermidis* DSMZ 20044	15.00 ± 0.00	19.00 ± 0.58	20.00 ± 0.00
*Salmonella infantis*	—	—	10.00 ± 0.00
*Salmonella kentucky*	—	7.00 ± 0.00	10.00 ± 0.00
*Salmonella typhimurium* SL1344	8.00 ± 0.58	—	10.00 ± 1.15
*Acinetobacter baumannii* MDR	—	10.00 ± 0.00	11.00 ± 0.00
*Proteus vulgaris* MDR	10.00 ± 1.15	14.00 ± 1.15	11.00 ± 1.15
*Streptococcus pneumoniae* MDR	10.00 ± 0.00	14.00 ± 1.15	13.00 ± 0.58

—: no inhibition.

**Table 4:** MIC values for *L. stoechas* (MIC values in *µ*g/mL).

	MIC
*Bacillus subtilis* DSMZ 1971	35.9
*Candida albicans* DSMZ 1386	35.9
*Enterobacter aerogenes* ATCC 13048	—
*Escherichia coli*	—
*Escherichia coli* ATCC 25922	—
*Enterococcus durans*	35.9
*Enterecoccus faecalis* ATCC 29212	35.9
*Enterecoccus faecium*	17.95
*Klebsiella pneumoniae*	—
*Listeria innocua*	35.9
*Listeria monocytogenes* ATCC 7644	35.9
*Pseudomonas aeruginosa* DSMZ 50071	35.9
*Pseudomonas fluorescens* P1	35.9
*Staphylococcus aureus*	17.95
*Staphylococcus aureus* ATCC 25923	17.95
*Salmonella enteritidis* ATCC 13076	35.9
*Staphylococcus epidermidis* DSMZ 20044	8.98
*Salmonella infantis*	—
*Salmonella kentucky*	35.9
*Salmonella typhimurium* SL1344	35.9
*Acinetobacter baumannii* MDR	35.9
*Proteus vulgaris* MDR	35.9
*Streptococcus pneumoniae* MDR	35.9

—: no inhibition.

## Data Availability

The data used to support the findings of this study are available from the corresponding author upon request.

## Abbreviations

GC-MS: Gas chromatography-mass spectrophotometry method.
